# The influence of learning interest on complex mathematical problem-solving ability: the mediating effect of classroom disruptive behavior and self-efficacy

**DOI:** 10.3389/fpsyg.2025.1638695

**Published:** 2025-10-16

**Authors:** Yongzhao Wang, Bingqing Xie, Lijun Zhou, Lisha Wang, Hua Jin

**Affiliations:** ^1^School of Mathematics and Statistics, Anyang Normal University, Anyang, Henan, China; ^2^School of Management and Economics, North China University of Water Resources and Electric Power, Zhengzhou, Henan, China; ^3^Department of Chinese Language and Literature, Gyeongsang National University, Jinju-si, Republic of Korea

**Keywords:** learning interest, classroom disruptive behavior, self-efficacy, complex mathematical problem-solving ability, mediating effect

## Abstract

**Introduction:**

The ability to solve complex mathematical problems has become a key indicator of students' mathematical literacy and innovative capacity.

**Methods:**

Based on the TIMSS 2023 data and focused on eighth-grade students in Taiwan, China, the United States, and Turkey, the study investigates the interaction mechanisms among students' learning interest (LI), classroom disruptive behavior (CDB), self-efficacy (SE), and complex mathematical problem-solving ability (CMPSA) through constructing a structural equation model.

**Results:**

LI, CDB, and SE are significantly correlated with CMPSA and can predict CMPSA. In addition, LI not only influences CMPSA directly, but also has an indirect effect through CDB and SE, including both parallel and chain mediation effects. Cross-cultural analysis further reveals significant regional differences in the impact mechanisms of CMPSA. To be specific, Taiwan, China is mainly characterized by the direct effect of LI, while the United States and Turkey rely on the indirect path of SE.

**Discussion:**

These explorations systematically reveal the formation mechanism of CMPSA from the perspective of multidimensional interaction of cognition, emotion, and environment, enrich the cross-cultural theoretical framework of CMPSA, and provide empirical basis for optimizing mathematics teaching practices and formulating regionally adaptive intervention strategies under different education systems.

## 1 Introduction

Since the early 21st century, mounting societal complexity has outgrown traditional competency frameworks, making complex problem-solving capacity the decisive benchmark of individual competitiveness in an innovation-driven era (Veríssimo et al., 2024). Mathematics, a core discipline that runs continuously from basic to higher education, serves both as an instrumental tool and a mode of thinking. On the one hand, it provides methodological support for the natural sciences, engineering technology, and even the social sciences. On the other hand, through abstraction, reasoning and modeling, it fosters logical thought and innovative awareness, making it widely regarded as one of the most representative vehicles for cultivating key competences. Complex mathematical problem-solving ability (CMPSA) emphasizes that learners must flexibly integrate mathematical concepts, cognitive strategies, and metacognitive resources in unstructured, open-ended, or interdisciplinary contexts to generate creative solutions through a complete process of systematic analysis, abstract modeling, reasoning and proof, and reflective validation (Guberman and Leikin, 2013). CMPSA aligns closely with the “four competencies” highlighted in China's curriculum standards—discovering, posing, analyzing, and solving problems—and constitutes a critical foundation for fulfilling the “practical innovation” dimension outlined in the Chinese Student Core Competencies Framework (Nasrulloh et al., 2023). Despite the widely recognized importance of CMPSA, there are still some pressing issues in the cultivation of relevant abilities. The prior research indicate that students generally excel at solving well-structured routine problems but show significant limitations and insufficient transfer abilities when dealing with complex and uncertain real-world situations (Woo and Falloon, 2022; Choi et al., 2014). Therefore, it is important to explore the factors influencing students' CMPSA to enhance their capabilities and nurture innovative talents. The formation of CMPSA is a multifaceted process, primarily shaped by an intricate interplay of cognitive, emotional, and environmental factors. Among them, self-efficacy (SE), learning interest (LI), and classroom disruptive behavior (CDB) are key influencing factors in the process of students' learning and development (Hidi and Renninger, 2006; Schiefele, 1991; Jennings and Greenberg, 2009; Sutherland and Oswald, 2005).

### 1.1 The relationship between self-efficacy and complex mathematical problem-solving ability

On the cognitive aspect, self-efficacy (SE) is defined as individuals' subjective belief and confidence in their ability to successfully complete specific mathematical tasks or achieve mathematical learning goals within the context of mathematics learning (Zakariya, 2022). It involves an assessment of one's own mathematical abilities, and expectations of effectively coping with and succeeding in the face of mathematical problems and learning challenges. SE is an important component of motivation in mathematics learning, influencing behavioral choices, effort, persistence, and emotional responses during the learning process (Pajares and Miller, 1994). Prior research has found that individuals with high SE tend to achieve better results in mathematics and are more willing to invest time and energy into learning mathematics (Hackett and Betz, 1989). Moreover, SE plays a significant role in stimulating learning motivation. Those with high SE have their intrinsic motivation fully activated, viewing mathematics learning as a challenge rather than a burden, and actively exploring the mysteries of mathematical knowledge (Stevens et al., 2006). The positive learning attitude enables them to better focus their attention and efficiently employ various learning strategies when facing mathematical learning tasks. When individuals believe in their ability to solve mathematical problems, they are more likely to persist in trying when encountering difficult problems, rather than giving up easily, thereby effectively enhancing their CMPSA (Marsh and Ayotte, 2003).

### 1.2 The relationship between learning interest and complex mathematical problem- solving ability

In the critical process of individual development, emotional factors play a pivotal role in students' abilities and should not be overlooked. Among them, LI refers to the comprehensive psychological state of students' positive emotional tendencies, sustained willingness to participate, and intrinsic motivational tendencies in mathematical activities. It can stimulate their active participation in the mathematics learning process, and arouse the curiosity and desire for knowledge in mathematics (Karwowski and Beghetto, 2019; Appiah et al., 2022). The non-cognitive factor is generally regarded as a key factor influencing problem-solving ability through pathways such as enhancing cognitive persistence, increasing error tolerance, and promoting deep processing. However, there is still theoretical disagreement regarding its relationship with CMPSA. Existing research has revealed a noteworthy contradiction. On the one hand, LI can significantly enhance cognitive engagement and persistence (Roche et al., 2023). On the other hand, Boggiano and Ruble (1979) mentioned the “over-motivation effect,” where individuals' intrinsic interest may be inhibited when they overly rely on extrinsic motivation (such as high-score rewards) in mathematics learning. Students who are driven solely by interest but lack feedback on their abilities may not perform as expected. This suggests that LI alone may not effectively translate into an advantageous performance in solving complex problems. Based on social cognitive theory, interest in mathematics can have a lasting impact on achievement outcomes. Students who are interested in mathematics often show higher achievement motivation in mathematics learning process, are willing to invest more time and energy in exploring mathematical problems, and thus achieve better results in mathematics (Yang et al., 2025). In addition, LI may also enhance SE, enabling them to maintain a positive attitude and sustained learning motivation when facing difficulties and challenges (Lin et al., 2020), thereby enhancing CMPSA.

### 1.3 The relationship between classroom disruptive behavior and complex mathematical problem-solving ability

Environmental factors, especially classroom order, also have a profound impact on students' mathematics learning. CDB is characterized by the actions that violate classroom discipline and disrupt normal teaching order during the teaching process (Zhu and Kaiser, 2022; Carrasco et al., 2022). These behaviors are typically manifested as talking out of turn, horseplay, playing with objects, or failing to follow classroom rules. They not only disrupt the normal order of the classroom and affect teaching progress and effectiveness, but also interfere with the attention of other students, reducing the overall learning atmosphere and efficiency (Colvin, 2010). From the perspective of classroom ecology theory, the classroom is a dynamic interactive system in which teacher behavior, student responses, and environmental factors together form an organic whole (Evertson and Weinstein, 2013). CDB is often indicative of both an individual student's behavioral issue and an imbalance in the classroom ecology. It is worth noting that moderate classroom interaction may help break mental stereotypes and stimulate creative thinking. However, continuous or excessive CDB can seriously disrupt cognitive coherence and interfere with the formation of deep mathematical thinking. Research shows that CDB not only distracts students' attention, causing them to miss key knowledge points and problem-solving strategies, thereby affecting the integrity of their mathematical knowledge structure, but may also reflect students' negative attitudes and lack of interest in mathematics learning process (Marder et al., 2023). The emotional state may further reduce their initiative to actively explore mathematical problems, decrease the degree of learning engagement, and diminish SE of the mathematical abilities (Ayotola and Adedeji, 2009). The decline in SE and the weakening of LI can form a vicious cycle that indirectly hinders the development of CMPSA. Tripon (2022)'s study showed that teachers, through professional training, can adopt more effective teaching strategies to improve classroom order, reduce CDB, and thereby promote students' cognitive participation and higher-order thinking development. Therefore, CDB may be an independent environmental variable and may also serve as an important mediating mechanism. Students with a higher LI often exhibit better behavioral norms, thereby reducing CDB. In turn, good classroom order helps students focus on learning, enhance SE, and ultimately promote the development of CMPSA (Byiringiro, 2024). The more frequent the CDB, the worse the students' academic performance tends to be, which indirectly confirmed the negative impact of CDB on students' mathematics learning (Liu and Huang, 2025).

The previous research on students' ability to solve problems primarily focuses on three dimensions. In the emotional aspect, control-value theory has confirmed that LI promotes deep cognitive processing through an emotional arousal mechanism (Pekrun, 2006). At the cognitive aspect, Bandura (1997)'s pioneering research has indicated that SE influences problem-solving strategy selection by regulating the allocation of cognitive resources. Environmentally, Osher et al. (2010) has investigated three methods for improving school discipline practices and student behavior, including an ecological approach to classroom management. Despite the valuable achievements of prior studies, there are still some gaps in the existing research that need to be further filled. First, few studies have examined the synergistic effects of the three categories of factors simultaneously, resulting in an insufficient understanding of the “LI-SE-CDB” triangular relationship. Second, the current research on CDB is sparse and largely lacks empirical evidence on how CDB affects students' learning outcomes, leaving the mechanisms unclear across contexts (Kalargiros and Manning, 2015; Nie and Liem, 2013). Finally, previous studies have mainly focused on small-scale research within the same region or country, with relatively scarce cross-cultural comparative studies. Therefore, to fill the above research gaps, the present study focuses on eighth-grade students from Taiwan, China, the United States, and Turkey in the TIMSS large-scale international assessment project, to systematically reveal the influencing mechanisms of CMPSA by integrating cognitive (SE), emotional (LI), and environmental (CDB) factors. The three regions have performed well in the TIMSS mathematics assessment, yet each has its own characteristics, representing Asia, North America, and Europe, respectively, and have comparable education systems and assessment data. These explorations could hopefully provide a new perspective and a more comprehensive framework for understanding the influencing mechanisms of students' CMPSA, offer more targeted guidance for mathematics education practice, enrich the understanding of mathematics learning characteristics in different cultural contexts, and propose valuable references for global education exchange and cooperation.

## 2 The present study

Previous studies have typically examined emotional (LI), cognitive (SE), and environmental (CDB) influences on CMPSA in isolation or in pairs, leaving the joint effects of all three factors largely unexplored. Consequently, the synergistic mechanisms among these dimensions remain insufficiently understood. There is currently no unified conclusion regarding the understanding of the role patterns of CDB. Furthermore, existing evidence relies predominantly on small, single-country samples, limiting cross-cultural comparability and generalizability. These gaps highlight the urgent need for large-scale, multi-national research that models the interrelationships among LI, SE, and CDB within a unified framework.

Building on the previous research, the current study, focused on eighth-grade students from Taiwan, China, the United States, and Turkey in TIMSS, analyzes the impact of factors such as LI, CDB, and SE on CMPSA and further explores the influencing mechanisms of CMPSA. Specifically, the research objectives of the study include: (1) examine how LI affects eighth-grade students' CMPSA; (2) explore whether CDB and SE have parallel mediating effects between LI and CMPSA; (3) analyze whether CDB and SE have chain mediating effects between LI and CMPSA; (4) investigate whether there are significant regional differences in the mechanisms influencing CMPSA.

**Figure 1:**
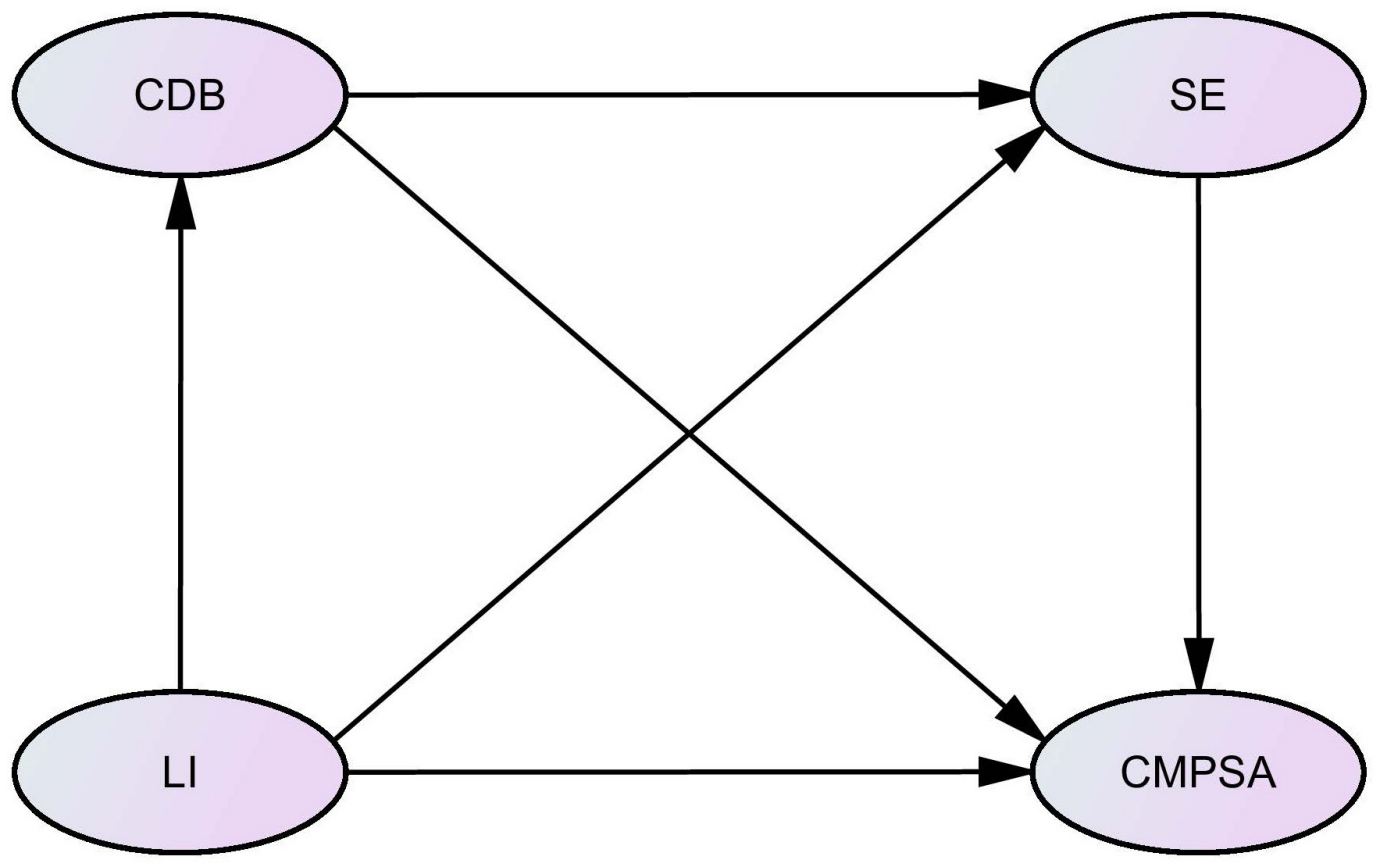
Hypothetical model.

The following hypotheses are proposed for this study. The first hypothesis (H1) posits that LI significantly and positively predicts CMPSA. The second hypothesis (H2) suggests that CDB and SE have parallel mediating effects between LI and CMPSA. The third hypothesis (H3) states that CDB and SE have chain mediating effects between LI and CMPSA. The fourth hypothesis (H4) emphasizes that there are significant regional differences in the mechanisms of CMPSA. The hypothetical model based on the above research hypotheses is shown in Figure 1.

## 3 Methods

### 3.1 Data sources

This research employs data from the “Trends in International Mathematics and Science Study” 2023, which focuses on eighth-grade participants. TIMSS, overseen by the International Association for the Evaluation of Educational Achievement (IEA), is a comprehensive international assessment that tracks the progress in mathematics and science learning among fourth and eighth-grade students globally. Since 1995, this project has been conducted every four years, with 8786 schools from 47 countries or regions (including three benchmark regions) participating in TIMSS 2023 (Mullis et al., 2021). The present study focuses on eighth-grade students in Taiwan, China, the United States, and Turkey, which each have their own unique characteristics in terms of educational philosophy, cultural traditions, and the level of mathematics education, providing a new perspective. The study sample includes 5,543 students from Taiwan, China, of whom 2,646 are female (47.7%) and 2,896 are male (52.2%); 8,074 students from the United States, of whom 3,946 are female (48.9%) and 4,128 are male (51.1%); and 4,925 students from Turkey, of whom 2,433 are female (49.4%) and 2,492 are male (50.6%).

### 3.2 Instruments

#### 3.2.1 Learning interest

The learning interest scale uses Question 19 from the TIMSS 2023 student context questionnaire to assess students' interest in mathematics. Upon conducting a detailed analysis and assessment of the data, the scale was optimized, retaining Questions A, D, E, F, G, H, and I from Question 19, ensuring the accuracy and validity of the scale. The scale uses a 4-point scoring system (1 = Strongly Agree, 4 = Strongly Disagree), with a representative item being “I enjoy learning mathematics.” These items are reverse-coded, with higher scores indicating stronger interest in mathematics. In the current study, the Cronbach's α coefficient for the scales was 0.938, indicating good reliability (Kline, 2023). The exploratory factor analysis showed that the factor loadings for all items were greater than 0.5, suggesting a close relationship between the items and the variables. The composite reliability (CR) was 0.938, and the average variance extracted (AVE) was 0.685, both of which are within the acceptable range (Fornell and Larcker, 1981). Additionally, the square root of the AVE was higher than the correlation coefficients between the variables, showing that the scales have good discriminant and convergent validity.

#### 3.2.2 Classroom disruptive behavior

The classroom disruptive behavior scale uses Question 21 from the TIMSS 2023 student context questionnaire to assess disruptive behaviors among students in the classroom. The scale selects Questions A, B, C, D, E, F, and G from Question 21, totaling six items. The scale uses a 4-point scoring system (1 = Every class, 4 = Never), with a representative item being “It takes my teacher a long time to quiet the class down.” These items are reverse-coded, with higher values indicating more disruptive behavior in the classroom. In this study, the scale's α coefficient was 0.913, indicating good reliability. Exploratory factor analysis revealed that the scales possess good structural validity. CR was 0.914, and AVE was 0.640, both of which are within the acceptable range. Moreover, the square root of the AVE was higher than the correlation coefficients between the variables, demonstrating that the scale has good discriminant and convergent validity.

#### 3.2.3 Self-efficacy

The self-efficacy scale uses Question 22 from the TIMSS 2023 student context questionnaire to assess students' self-efficacy. Upon conducting a detailed analysis and assessment of the data, the scale was optimized, retaining Questions B, C, G, and H from Question 22, ensuring the accuracy and validity of the scale. The scale uses a 4-point scoring system (1 = Strongly Agree, 4 = Strongly Disagree), with higher scores indicating higher self-efficacy. A representative item is “Mathematics is not my strong suit.” The scale's α coefficient for the present study was 0.856, indicating acceptable reliability. Exploratory factor analysis showed that the factor loadings for all items were greater than 0.5, suggesting a close relationship between the items and the variables. CR was 0.856 and AVE was 0.599, both of which are within the acceptable range. Additionally, the square root of the AVE was higher than the correlation coefficients between the variables, indicating that the scale has good discriminant and convergent validity. Furthermore, the heterotrait-monotrait ratio of correlations (HTMT) test revealed that the HTMT values between the variables were all less than 0.9, demonstrating good discriminant validity among the variables (Henseler et al., 2015).

#### 3.2.4 Complex mathematical problem-solving ability

The study employs five plausible values (PVs) each for mathematical application and mathematical reasoning from the TIMSS assessment to measure students' CMPSA. In the TIMSS survey, PVs are not the students' raw scores. Instead, they are the actual ability distribution values of the participants calculated through Item Response Theory (IRT), taking into account students' background variables such as gender, grade, and social background, and then randomly drawn as the students' possible ability values (Wu, 2005). These PVs reflect the potential range of students' abilities, but it is not appropriate to simply average the PVs to estimate individual students' performance. Therefore, the current study takes the average of these two types of PVs as the outcome variable to comprehensively evaluate the ability of eighth-grade students in solving complex mathematical problems.

#### 3.2.5 Control variables

Research has shown that students of different genders and ages may differ in their CMPSA (Ramírez-Uclés and Ramírez-Uclés, 2020). Therefore, gender and age, which may influence the variables discussed in this study, including LI, CDB, SE, and CMPSA, should be controlled.

### 3.3 Data processing

The TIMSS employs a two-stage stratified cluster random sampling method. In the first stage, schools are selected as units, using the roster of eighth-grade schools provided by the National Research Center (NRC) as the sampling frame. Schools are first stratified by explicit variables such as country/region and school type, then sorted by implicit variables such as school size and academic performance. Probability Proportional to Size (PPS) sampling combined with systematic random sampling is used to select schools, with two backup schools designated for each sample school. In the second stage, targeting the class and student levels, the WinW3S software developed by IEA is used to select one or more entire classes from all eighth-grade classes with equal probability systematic random sampling. All students in the selected classes participate in the test. If the class size is too small, they are combined into a “pseudo-class” for sampling. Additionally, this study uses SPSS for preliminary data processing, statistics, and analysis, and AMOS for testing the hypothesized mediating effects, calculating the bias-corrected 95% confidence intervals for the estimates, and performing 5000 bootstrap replications. If the confidence interval does not include zero and the significance test *p* ≤ 0.05, the effect is considered significant. All analyses are weighted using student sampling weights to ensure the representativeness of the overall study sample.

### 3.4 Common method bias

The Harman single-factor test was utilized to assess potential common method bias across all items pertaining to LI, CDB, SE, and CMPSA. The findings revealed four factors with eigenvalues exceeding 1. The most significant factor explained 33.845% of the variance, which is beneath the established cutoff of 40%. Consequently, it can be inferred that common method bias has a minimal influence on the outcomes of the study.

## 4 Results

### 4.1 Descriptive analysis

To analyze the current status of students' LI, CDB, SE, and CMPSA, descriptive statistics were calculated, including mean (*M*), standard deviation (*SD*), skewness, and kurtosis. The results are as presented in Table 1. It can be seen that the *M* of LI range from 2.26 to 2.79, with *SD* between 1.00 and 1.15, indicating some variability in students' interest in mathematics learning. The *M* of CDB range from 2.23 to 2.59, with *SD* between 0.98 and 1.12, suggesting differences in classroom behavior among students, but overall, the frequency of disruptive behavior is at a moderate level. The *M* of SE range from 2.42 to 2.71, with *SD* between 1.03 and 1.13, reflecting some fluctuations in students' confidence in their mathematical abilities. For CMPSA, the *M* for mathematical application and reasoning are 495.91 and 493.33, respectively, with *SD* of 102.00 and 100.74, indicating some differences in students' performance in these two aspects, but overall, the ability levels are relatively close. These data results suggest that students show potential in multiple aspects of mathematics learning, but also exhibit significant differences and issues that require further investigation into the relationships between these factors.

**Table 1:** Descriptive statistics.

**Variable**	**Item**	***M***	***SD***	***Skewness***	***Kurtosis***
Learning interest	LI1	2.67	1.05	−0.31	−1.09
	LI2	2.79	1.00	−0.39	−0.88
	LI3	2.57	1.08	−0.16	−1.23
	LI4	2.29	1.03	0.25	−1.09
	LI5	2.42	1.07	0.06	−1.24
	LI6	2.26	1.05	0.25	−1.16
	LI7	2.30	1.15	0.23	−1.39
Classroom disruptive behavior	CDB1	2.59	0.98	0.10	−1.04
	CDB2	2.36	1.02	0.29	−1.01
	CDB3	2.35	1.06	0.30	−1.12
	CDB4	2.38	1.08	0.26	−1.19
	CDB5	2.31	1.05	0.37	−1.02
	CDB6	2.23	1.12	0.41	−1.18
Self-efficacy	SE1	2.58	1.07	−0.10	−1.20
	SE2	2.42	1.12	0.10	−1.34
	SE3	2.56	1.13	−0.08	−1.36
	SE4	2.29	1.03	0.27	−1.06
Complex mathematical problem-solving ability	APP	495.91	102.00	0.11	−0.50
	REA	493.33	100.74	0.13	−0.41

### 4.2 Correlation analysis

To investigate the underlying mechanisms, the Pearson correlations between LI, CDB, SE, and CMPSA were further examined. The analysis results, as presented in Table 2, reveal significant correlations among all four variables. To be specific, LI and SE are significantly and positively correlated with CMPSA (*r* = 0.240, 0.364), indicating that higher levels of LI and SE are associated with stronger CMPSA. Meanwhile, CDB is significantly and negatively correlated with SE (*r* = −0.158) and CMPSA (*r* = −0.159), suggesting that CDB can undermine students' SE and CMPSA. Additionally, a significant positive correlation exists between LI and SE (*r* = 0.440). These findings suggest that enhancing students' LI and SE and reducing CDB can contribute to improving their CMPSA.

**Table 2:** Correlation analysis among major study variables.

**Variable**	**LI**	**SE**	**CDB**	**CMPSA**
LI	1			
SE	0.440^**^	1		
CDB	−0.062^**^	−0.158^**^	1	
CMPSA	0.240^**^	0.364^**^	−0.159^**^	1

^**^p < 0.01.

### 4.3 Multiple linear regression analysis of complex mathematical problem-solving ability

A comprehensive examination of the assumptions was performed to ensure that the data met the fundamental conditions for multiple linear regression analysis. These conditions include the linear relationship between the dependent and independent variables, the independence of residuals, the normality of residuals, homogeneity of variance, and the absence of multicollinearity among independent variables. Given that these conditions were satisfied, the present study conducted multiple linear regression analysis with students' CMPSA as the dependent variable and LI, CDB, SE, gender, and age as independent variables, with results as presented in Table 3. It is evident that LI (β = 0.098) and SE (β = 0.299) significantly and positively predicted CMPSA, while CDB (β = −0.107) significantly and negatively predicted the ability. To further examine the mechanisms through which LI, CDB, and SE affect CMPSA, subsequent analyses were conducted with CDB and SE as mediating variables.

**Table 3:** Multiple linear regression analysis among major study variables.

**Variable**	***B***	***SE***	**β**	***t* value**
LI	10.880	0.053	0.098	205.903^***^
SE	33.105	0.053	0.299	621.770^***^
CDB	−12.261	0.050	−0.107	−247.365^***^
Gender	6.810	0.087	0.034	78.604^***^
Age	−24.349	0.096	−0.109	−253.682^***^
Constant	749.219	1.372		546.083^***^

^***^p < 0.001.

### 4.4 Mediating effects of classroom disruptive behavior and self-efficacy

Structural equation models (SEM) were constructed to incorporate CDB and SE as mediating variables in the relationship between LI and CMPSA. Both models fit the data well. Specifically, for the model with CDB as the mediating variable: χ^2^(87) = 1, 479, 804.079, CFI = 0.975, NFI = 0.975, TLI = 0.969, RMSEA = 0.061. For the model with SE as the mediating variable: χ^2^(62) = 1, 683, 596.952, CFI = 0.967, NFI = 0.967, TLI = 0.958, RMSEA = 0.077, indicating a good fit between the data and the models (Hu and Bentler, 1999). The results, as detailed in Table 4, show that CDB has a mediating effect value of 1.086, accounting for 4.14% of the total effect, while SE has a mediating effect value of 18.296, accounting for 68.85% of the total effect, with neither confidence interval including zero. Therefore, both CDB and SE partially mediate the relationship between LI and CMPSA, confirming the existence of parallel mediation effects.

**Table 4:** Mediating effect analysis.

**Path**		**Effect**	**95% CI lower**	**95% CI upper**	***p***	**Effect %**
LI → CDB → CMPSA	Total effect	26.183	26.091	26.282	^***^	
	Direct effect	25.098	25.005	25.195	^***^	95.86%
	Indirect effect	1.086	1.069	1.102	^***^	4.14%
LI → SE → CMPSA	Total effect	26.575	26.485	26.673	^***^	
	Direct effect	8.278	8.158	8.405	^***^	31.15%
	Indirect effect	18.296	18.221	18.373	^***^	68.85%

^***^p < 0.001.

### 4.5 Chain mediating effect analysis of classroom disruptive behavior and self-efficacy

**Figure 2:**
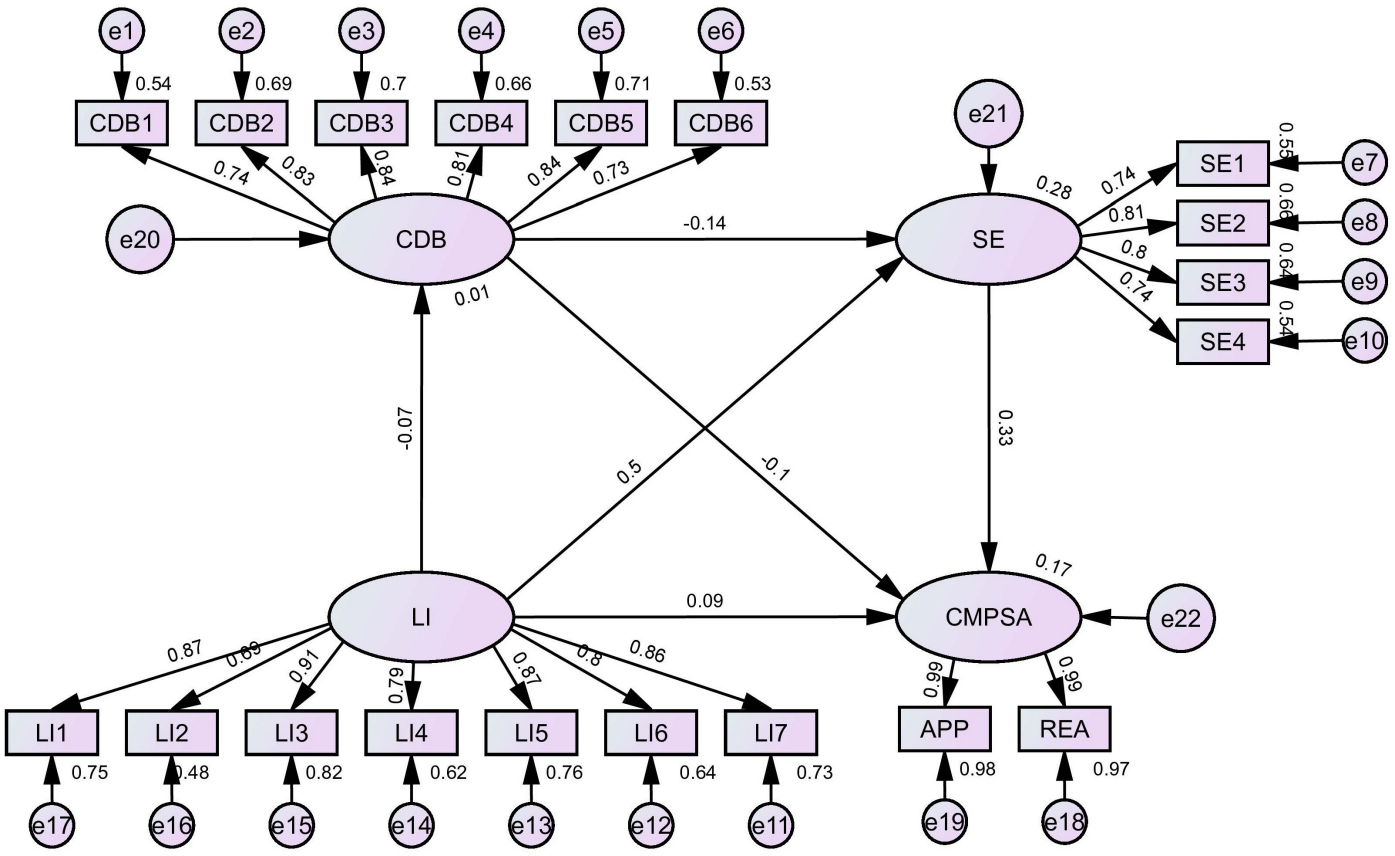
Path diagram for the hypothetical structural equation model.

CDB and SE were incorporated as mediating variables in the relationship between LI and CMPSA using AMOS to construct SEM, thereby examining the chain mediating effects of CDB and SE, with results as showed in Table 5 and Figure 2. The model fit indices were satisfactory: χ^2^(146) = 2355036.194, CFI = 0.966, NFI = 0.966, TLI = 0.960, RMSEA = 0.059. These indices all fall within the threshold range for a well-fitting model, providing strong support for the reliability of the research results. It can be seen that the total indirect effect size was 17.940, accounting for 67.61% of the total effect. Specifically, the indirect effect of LI → CDB → CMPSA was 0.751 (2.83% of the total effect), the indirect effect of LI → SE → CMPSA was 16.859 (63.53% of the total effect), and the chain mediation path (LI → CDB → SE → CMPSA) had an indirect effect of 0.330 (1.24% of the total effect). The 95% confidence intervals for all three paths did not include zero. In summary, the chain mediating roles of CDB and SE in the relationship between LI and CMPSA were confirmed.

**Table 5:** Chain mediating effect analysis of classroom disruptive behavior and self-efficacy.

**Path**	**Effect size**	**95% CI lower**	**95% CI upper**	***p***	**Effect %**
Total effect	26.536	26.446	26.633	^***^	
Direct effect: MI → CMPSA	8.595	8.478	8.719	^***^	32.39%
Total indirect effect	17.941	17.866	18.017	^***^	67.61%
Path 1	0.751	0.739	0.764	^***^	2.83%
Path 2	16.859	16.784	16.934	^***^	63.53%
Path 3	0.330	0.325	0.336	^***^	1.24%

^***^p < 0.001; Path 1 is LI → CDB → CMPSA; Path 2 is LI → SE → CMPSA; Path 3 is LI → CDB → SE → CMPSA.

### 4.6 Regional differences in the mechanism influencing complex mathematical problem- solving ability

To further investigate whether the mechanisms underlying CMPSA vary across different countries or regions, this subsection conducted an in-depth analysis of mechanism differences among Taiwan, China, the United States, and Turkey.

#### 4.6.1 Regional variance analysis of complex mathematical problem-solving ability

The separate mediation analyses were conducted for the three regions, with the results as reported in Table 6. Significant regional differences emerge in the mediating roles of CDB and SE between LI and CMPSA. On the one hand, although the total effect is significant in all three regions (*p* < 0.001), its magnitude differs markedly. Taiwan, China exhibits the largest total effect (39.249), followed by Turkey (36.055) and the United States (23.037), indicating that the overall impact of LI on CMPSA is strongest among students. On the other hand, the direct effect is significant and accounts for the highest proportion in Taiwan, China (51.15%), implying that LI primarily influences CMPSA through the direct path. By contrast, the United States and Turkey show larger proportions of total indirect effects (75.15% and 76.16%, respectively), with SE exerting the largest mediating influence (68.50% and 74.47%, respectively). Thus, in these two countries, LI mainly affects CMPSA via the mediating pathway of SE. Although the indirect effect is relatively smaller in Taiwan, China, it remains an important route. These discrepancies likely reflect heterogeneity in educational systems regarding the mechanisms of students' mathematical learning.

**Table 6:** Chain mediating effects of the three regions.

**Path**	**Taiwan, China effect size (% of effect)**	**The United States effect size (% of effect)**	**Turkey effect size (% of effect)**
Total effect	39.249^***^	23.037^***^	36.055^***^
Direct effect	20.074^***^ (51.15%)	5.725^***^ (24.85%)	8.597^***^ (23.84%)
Total indirect effect	19.175^***^ (48.85%)	17.312^***^ (75.15%)	27.458^***^ (76.16%)
Path 1	−0.235^***^ (−0.59%)	1.082^***^ (4.70%)	0.365^***^ (1.01%)
Path 2	19.388^***^ (49.39%)	15.780^***^ (68.50%)	26.850^***^ (74.47%)
Path 3	0.022^***^ (0.05%)	0.450^***^ (1.95%)	0.243^***^ (0.67%)

^***^p < 0.001.

#### 4.6.2 Regional differences analysis of complex mathematical problem-solving ability across contexts

Although the mechanism underlying CMPSA currently differs significantly across regions, whether these discrepancies originate from distinct mathematical competencies (i.e., mathematical application vs. mathematical reasoning) remains unclear. Consequently, this part re-examined regional differences in CMPSA by separately using mathematical application and mathematical reasoning as indicators. The analysis results are presented in Tables 7, 8, yielding the following findings. First, the influence of LI on CMPSA among students in Taiwan, China operates predominantly through the direct effect, which accounts for 62.7% (mathematical application) and 56.7% (mathematical reasoning) of the total effect, indicating that LI is directly converted into CMPSA. The chained indirect path (Path 3) involving CDB and SE contributes almost nothing, implying that students rely minimally on the chain mediation and depend mainly on SE. Second, the results show that mediation plays a critical role in mathematical competency development in the United States. The total indirect effect accounts for 71.9% (mathematical application) and 78.6% (mathematical reasoning), demonstrating that American students rely heavily on the mediating roles of CDB and SE when transforming LI into CMPSA. Although the effect size of Path 3 is small, its mediating mechanism remains significant. Finally, Turkey exhibits a pattern similar to that of the United States, with high total indirect effects of 78.5% (mathematical application) and 74.7% (mathematical reasoning), underscoring the importance of mediation. However, the effect size of Path 3 is limited at only 0.7%, suggesting that the immediate impact of the chained mediation path is modest. In summary, across different mathematical-competency contexts the mediating mechanisms through which LI is converted into CMPSA differ significantly among the three regions.

**Table 7:** The chain mediating effect of the three regions under mathematical application.

**Pathway**	**Taiwan, China effect size (% of effect)**	**The United States effect size (% of effect)**	**Turkey effect size (% of effect)**
Total effect	39.653^***^	24.132^***^	35.250^***^
Direct effect	24.861^***^ (62.7%)	6.779^***^ (28.1%)	7.576^***^ (21.5%)
Total indirect effect	14.792^***^ (37.3%)	17.353^***^ (71.9%)	27.673^***^ (78.5%)
Path 1	−0.176^***^ (−0.4%)	1.151^***^ (4.8%)	0.347^***^ (1.0%)
Path 2	14.951^***^ (37.7%)	15.753^***^ (65.2%)	27.081^***^ (76.8%)
Path 3	0.017^***^ (0.0%)	0.449^***^ (1.9%)	0.246^***^ (0.7%)

^***^p < 0.001.

**Table 8:** The chain mediating effect of the three regions under mathematical reasoning.

**Pathway**	**Taiwan, China effect size (% of effect)**	**The United States Effect size (% of effect)**	**Turkey effect size (% of effect)**
Total effect	40.690^***^	22.518^***^	36.382^***^
Direct effect	23.073^***^ (56.7%)	4.824^***^ (21.4%)	9.204^***^ (25.3%)
Total indirect effect	17.617^***^ (43.3%)	17.695^***^ (78.6%)	27.178^***^ (74.7%)
Path 1	−0.213^***^ (−0.5%)	1.041^***^ (4.6%)	0.374^***^ (1.0%)
Path 2	17.810^***^ (43.8%)	16.192^***^ (71.9%)	26.562^***^ (73.0%)
Path 3	0.020^***^ (0.0%)	0.462^***^ (2.1%)	0.241^***^ (0.7%)

^***^p < 0.001.

## 5 Discussion

The present study investigates how LI affects students' CMPSA and examines the mediating roles of CDB and SE within the relationship. It uncovers both the direct impact of LI on CMPSA and the indirect effects that operate through behavioral and psychological pathways, yielding four valuable findings.

The first finding demonstrates that LI exerts a significant positive effect on CMPSA, confirming H1. The finding aligns with the core perspective of self-determination theory, which suggests that intrinsically motivated learning driven by interest can facilitate deeper cognitive processing (Deci and Ryan, 2012). Additionally, cognitive neuroscience research indicates that the positive effect may stem from interest's reshaping of the brain's information processing system (Popescu et al., 2019). Especially, fMRI research emphasizes that when individuals engage with mathematical problems they find interesting, the functional connectivity between the prefrontal cortex (responsible for higher cognitive functions) and the hippocampus (involved in memory encoding) is significantly enhanced (Dixon and Christoff, 2014). The neural integration not only improves working memory efficiency, but also promotes deeper problem-solving processing—key cognitive processes required for solving complex mathematical problems (Rushworth et al., 2011). When students develop a stable individual interest in mathematics, it generates a sustained intrinsic motivation, which encourages them to actively seek out challenging mathematical problems, and enables them to demonstrate greater persistence and resilience when facing difficulties (Rodríguez et al., 2021). Therefore, LI is not merely a psychological inclination, but a systemic factor that reshapes the brain's information processing methods. It optimizes the allocation of cognitive resources and the efficiency of neural networks, ultimately enhancing an individual's ability to solve complex mathematical problems.

The second finding corroborates H2 by confirming that CDB and SE function as parallel mediators between LI and CMPSA. On the one hand, CDB exerts a significant partial mediation effect, accounting for 4.14% of the total effect. The result aligns with classroom ecology theory, which posits a continuous bidirectional interplay between student behavior and the classroom environment (Evertson and Weinstein, 2013). Students with higher LI typically display greater engagement and adaptive conduct, thereby buffering the negative impact of CDB and indirectly fostering CMPSA. Nevertheless, even after controlling for LI, CDB retains an independent negative predictive power on achievement, plausibly through three mechanisms. First, disruptive incidents consume limited cognitive resources, especially working-memory capacity, thereby reducing the pool available for higher-order thinking (Webb, 2009). Second, instructional interruptions caused by disorder lead to the omission of key knowledge points, undermining the coherence of students' knowledge structures (Emmer and Sabornie, 2015). Third, CDB of a few students may evoke collective anxiety, further depressing overall learning outcomes (Reyes et al., 2012). Although the effect size of the mediating path is relatively small, it indicates that educators should pair interest-enhancement strategies with effective classroom management to attenuate environmental distractions and cultivate an ecological climate conducive to deep learning. On the other hand, SE exerts a far stronger mediating influence, explaining 68.85% of the total effect and illuminating the psychological mechanism whereby LI affects CMPSA. The finding extends social cognitive theory's explanatory power within mathematics education (Bandura, 2001). According to the framework, SE functions as the core mechanism of human agency, bridging interest-driven motivation and cognitive performance. Specifically, heightened LI prompts students to engage more actively in mathematical activities, seek challenging tasks, and accumulate mastery experiences, thereby markedly strengthening their “I can” beliefs (Hwang and Oh, 2021). Enhanced SE motivates learners to approach more demanding problems in turn, deploy deep cognitive strategies (e.g., elaboration and metacognitive regulation), and display greater resilience and emotional control when difficulties arise (Zimmerman, 2000). Additionally, Zhang and Wang (2020) demonstrated that SE serves as a critical bridge between affective factors and cognitive performance in mathematics, and Usher and Pajares (2008) showed that efficacious students are more inclined to use advanced strategies and persist longer. Consequently, fostering SE represents a pivotal educational intervention for converting interest into tangible competency.

The present study is the first to validate the chain-meditation pathway “LI → CDB → SE → CMPSA,” yielding the third major finding and confirming H3. Integrating behaviorist and cognitive-psychology perspectives, the pathway transcends single-theory limitations by modeling an external-to-internal cascade that culminates in advanced cognitive performance. To be specific, intrinsic motivation (LI) initiates constructive study behavior and suppresses classroom disordered conduct (Ryan and Deci, 2000b). CDB, acting as a chronic environmental stressor, erodes SE not only by delivering repeated failure experiences, but also via neurobiological mechanisms (amygdala hyper-activation coupled with prefrontal inhibition) that directly compete for the cognitive resources required by complex problem solving (Arnsten, 2009). Diminished SE subsequently attenuates the magnitude, intensity, and generality of students' motivation, strategic deployment, and persistence when confronting complex tasks, ultimately lowering overall competence. The effect size is small, yet this is typical for multi-step mediation models (Preacher and Hayes, 2008). Its statistical significance remains critical because it identifies the formation mechanism of a cognition-environment vicious cycle and provides a systemic target for educational interventions: motivation cultivation, classroom environment optimization, and adaptive efficacy belief construction must all be addressed simultaneously to foster higher-order ability.

The fourth finding corroborates H4 through cross-region comparisons among Taiwan, China, the United States, and Turkey, revealing pronounced regional heterogeneity in the formation mechanism of CMPSA that mirrors distinct educational cultures and instructional practices. Specifically, the magnitude of the indirect effect of LI on CMPSA differs significantly across the three regions. Students in Taiwan, China rely more heavily on the direct pathway, with LI accounting for over 50% of the total effect on CMPSA. Although SE plays a certain role, the influence of the classroom environment remains relatively small. The pattern reflects Taiwan's knowledge-centered, highly disciplined and examination-oriented system, whose efficient standardized pedagogy secures solid knowledge acquisition (Gao, 2014). However, the strong direct route may constrain students' capacity to application and reasoning in complex contexts, and simultaneously attenuates the mediating role of classroom interaction and affective factors. In contrast, the sample of the United States exhibits an overwhelmingly indirect pathway (total indirect effect >75%), with SE functioning as the core mediator, reflecting an instructional culture that prioritizes psychological construction, classroom interaction, and autonomous learning while emphasizing individual feelings and interactive quality (Dewey, 1986; Darling-Hammond, 2004). Moreover, the relative weakening of direct instruction may compromise the consolidation of foundational knowledge. Turkey resembles the United States in being indirect-dominant, with SE again the most prominent mediator, indicating a system heavily reliant on intrinsic motivation amid comparatively limited classroom-management structures and external support. Under the prevailing traditional pedagogy and potentially unequal educational resources, SE has become the pivotal driver of mathematics learning. Therefore, strategies to enhance students' CMPSA must be context-specific. In Taiwan, China, it is recommended that efficient direct instruction be retained while integrating inquiry-based and project-based learning to strengthen application and reasoning skills, concurrently optimizing the classroom climate. In the United States, foundational training and direct teaching should be reinforced within existing efficacy-centered practices to improve learning efficiency. In Turkey, classroom management and equitable resource allocation should be improved, and teachers' capacity to cultivate students' SE should be enhanced, so that LI can be converted into tangible competence. In summary, each region should leverage existing strengths and remedy identified weaknesses to achieve more balanced and effective mathematics education development.

The study offers the following principal contributions. First, the study overcomes the limitations of previous research that focused solely on a single dimension (cognitive, emotion, or environment). It is the first to incorporate LI (emotion), SE (cognitive), and CDB (environment) into a unified analytical framework. By constructing and validating the “Interest-Environment-Cognition” triangular interaction model, the current research addresses the insufficiency in the existing literature regarding the mechanisms of multi-factor synergistic effects. It reveals the multi-path formation mechanisms of CMPSA from a systemic perspective, thereby enriching the theoretical construction in the field of mathematics education. Second, empirical evidence clearly reveals that the mechanisms by which CDB affects CMPA, underscoring the critical role of learning environments in cultivating learners' abilities. Third, drawing on the large-scale TIMSS database, the current study conducts cross-cultural analyses of Taiwan, China, the United States and Turkey, thereby confirming the universality of the mediation model while revealing contextual heterogeneity in its operative pathways. Finally, the present research provides the first empirical validation of the chain pathway “LI → CDB → SE → CMPSA”, which integrates the core principles of behaviorism and social cognitive theory, accurately depicting the transformation process from external behavior to internal belief and then to competence, offering significant theoretical innovation and practical guidance.

Despite the valuable contributions, it has some limitations. First, the cross-sectional design captures statistical associations but precludes causal inference. For example, although the model posits that LI influences SE and CMPSA, successful problem-solving experiences may also enhance both SE and LI. Longitudinal or experimental designs are needed to test causal ordering and dynamic processes. Second, the data rely heavily on students' self-reports of LI, SE, and CDB. Although TIMSS instruments exhibit sound reliability and validity, self-report data are vulnerable to social-desirability bias, recall error, and culturally varying response styles. Systematic differences in the interpretation of “LI” or “CDB” may obscure true cross-group differences. Future studies should incorporate multiple informants (e.g., teacher ratings, classroom observations, behavioral logs) to enhance ecological validity and measurement accuracy. Third, although Taiwan, China, the United States, and Turkey provide representation from East Asia, North America, and Europe, the sample omits Africa, Latin America, and South Asia, limiting global generalizability. Finally, the “LI-CDB-SE” mediation model does not include potentially salient contextual variables such as teaching style, family support, peer interaction, or school climate, nor does it employ hierarchical linear modeling (HLM) to account for nested data structures. These contextual factors may interact across levels with CDB and SE to shape CMPSA development. Because the present study focuses on individual-level mechanisms and is constrained by data structure, HLM was not applied. Future research is recommended to incorporate macro-, meso-, and micro-level variables simultaneously and employ multilevel analysis methods such as HLM when constructing more integrated multilevel theoretical models, so as to provide more comprehensive and accurate theoretical explanations and inferences.

## 6 Conclusion

The current study aims to explore the impact of LI on CMPSA and to test the mediating role of CDB and SE. The main conclusions of the study are as follows: (1) LI, CDB, and SE significantly predict CMPSA. (2) CDB and SE mediate the relationship between LI and CMPSA, with both parallel and chain mediation effects identified. (3) The mechanism underlying CMPSA shows significant regional differences.

Based on the above conclusions, the following recommendations for improvement are proposed. (1) Employing effective teaching methods to cultivate learning interest. Novelty is a powerful catalyst for sparking students' intrinsic motivation to explore and, consequently, a highly effective means of fostering their interest in learning (Ryan and Deci, 2000a). Teachers should meticulously take into account a variety of factors when selecting and applying teaching methods, which include the characteristics of the mathematics subject, students' capacity to absorb knowledge, teachers' own qualifications (such as professional proficiency, practical experience, and personal traits), the resources provided by the school, and the constraints of teaching time. Additionally, it is crucial for teachers to closely monitor students' learning status, encourage them to question, and even deliberately make mistakes to allow students to correct them, which can yield surprising results. Furthermore, teachers should give students the autonomy to participate in teaching activities, to independently delve into problems, and to conduct exploratory attempts, thereby enabling students to truly become the masters of the classroom and to perceive learning as their own responsibility. (2) Updating classroom management concepts to establish a correct student perspective. Teachers, as the main body of classroom management, should promptly update their management concepts and transform them into conscious behaviors in educational practice (Reeve, 2006). Teachers should regard themselves as equal participants in dialogue with students during the teaching process, establish a democratic management mechanism, and accurately balance democratic management with maintaining classroom discipline. They should avoid autocratic and dictatorial management styles and punitive measures that may disrupt classroom harmony. At the same time, teachers should acknowledge and respect the differences among students to promote the comprehensive development of each student. (3) Integrating metacognitive strategies to enhance self-efficacy. SE is a crucial psychological factor for students when facing mathematical challenges, directly affecting their confidence and perseverance in problem-solving (Zimmerman, 2002). Integrating metacognitive strategies into teaching can significantly boost students' SE. Teachers can introduce metacognitive training to help learn to plan problem-solving steps (such as setting goals), monitor their thinking processes (such as adjusting strategies), and evaluate outcomes (such as reflecting on mistakes), thereby enhancing their sense of control over their abilities. In addition, teachers can provide students with tiered practice and immediate feedback to help them accumulate successful experiences and gradually tackle more complex problems. By integrating metacognitive strategies, students can not only enhance their SE, but also demonstrate greater resilience and creativity when solving complex mathematical problems.

## Data Availability

The original contributions presented in the study are included in the article/supplementary material, further inquiries can be directed to the corresponding author.
